# Pharmacopœia Homœopathica

**Published:** 1834-07-01

**Authors:** 


*Pharmacopœia Homœopathica.* Edidit T. F. QUIN, M.D., Medicus Ordinarius Leopoldi Primi Regis Belgarum, &c.—Londini, M.DCCC.XXXIV. 8vo. pp. 165.

WE recollect a remarkably instructive case in Dr. Paris's Treatise on Diet, which is quite apropos to the doctrine of Homœopathy. A clerk was in the habit of remaining at his office from nine to five, and then taking exercise for two hours, without refreshment. So long a fast, and so much work, had debilitated, and almost destroyed his digestive powers, and he sought the advice of the excellent physician just mentioned. Dr. Paris, after having administered medicine without benefit, made more particular inquiry into his patient's habits, and ordered him to take a lunch with him to his office. He did so, and got well. Now, no rational person can doubt that he would also have got well had he taken the tincture of belladonna, or of aconite, in the dose of " glob. ij. vel iij. trigesimæ attenuationis;" or that Homœopathy depends for its fame on the fact, that in chronic, or, as Hahnemann pleasantly calls them, *psoric* diseases, we have few or no specifics; and that the dietetic doctor will patch up more shattered constitutions than he who puts his trust in blue-pill and gentian.

Dr. Quin writes in the gravest of all languages, and, though we cannot compliment him on his Latinity, his gravity is really enviable. Far from ever allowing a nascent smile to betray the fact that what he is talking about is fudge, he keeps up the stern composure of a comic actor, who does not join in the laughter which he excites. He prepares his tinctures and powders, his ghosts of departed medicines, with the wholesome earnestness of a child building card-houses: indeed this is carried almost too far: for, as Montaigne tells us, that the sports of children are not to them sports, but are to be judged in them as their most serious actions, so one might be tempted to think that Dr. Quin had taken himself in, and would really treat cases in which physic might be of advantage, with his imaginary quantities, his medicinal differentials.

We will now indulge our readers with one of the formulæ of this most humorous Pharmacopœia.

ACONITUM NAPELLUS L.

*Triginta attenuationes* (X).

"Floret mensibus Julio et Augusto, quo tempore colligenda est.

"Succum e recente herba expressum cum pari quantitate Spiritus Vini misce, et viginti quatuor horis præterlapsis liquorem limpidum et clarum defunde.

"Unam hujus guttam cum nonaginta novem guttis Spiritus Vini bis agita, et signo (1) inscribe. Deinde attenua ad dilutionem trigesimam.

Dosis:—Glob. ij. vel iij. vigesimæ quartæ in morbis chronicis, in acutis vero tricesimæ attenuationis.

"Duratio:—Per duodequinquaginta horas.

"Antid.—Acida plantarum vel Vinum." (P. 36.)

The juice of aconite is to be mixed with an equal quantity of spirit of wine; one drop of the mixture is then to be diluted with ninety-nine drops of spirit, constituting the first attenuation; one drop of this again is to be diluted with ninety-nine drops of spirit, and this constitutes the second attenuation, and so on. A drop of the last mixture will have only $\overline{(1,000,000)^{10}}$th of the strength of a drop of the original compound of juice and spirit. In chronic diseases we may be so bold as to use the medicine only twenty-four times attenuated; in which state, however, it does not seem to us likely to produce very severe symptoms of narcotism; but we must not suppose that a whole drop is to be given for a dose: such rashness would make every hair stand erect on homœopathic heads. No; the words glob. ij. vel iij. refer to a much nicer subdivision. People often talk of a drop in the ocean; but in homœopathic practice, we learn to see an ocean in a drop. It is only in very few diseases, and to very stout men, says Dr. Quin, that a whole drop, or even half a drop, of any remedy can be given as a dose, and we are therefore to get the "globuli minimi" made of sugar and starch by the pastrycooks, of which about two hundred weigh a grain, and soaking these in the required tincture, we are to keep them for use. What these "globuli minimi" may be, we know not; our first guess was *sugar-plums;* but, though we have all eaten and given away sugar-plums (even reviewers do sometimes,) we never saw any weighing only the $\frac{1}{200}$th of a grain. The notation by which the degree of attenuation is indicated is ingenious enough.

1 signifies the first attenuation, and shows that the mixture denoted by it has, in a hundred drops, but one drop of the original compound of juice and spirit;

2 signifies the second attenuation, and shows that the mixture denoted by it has, in ten thousand drops, but one drop of the original compound;

I (the Roman numeral) signifies the third attenuation, and shows that the mixture denoted by it has, in a million drops, but one drop of the original compound;

II (the Roman numeral) signifies the sixth attenuation, and shows that the mixture denoted by it has, in a billion (1,000,000,000,000) drops, but one drop of the original compound;

—and so on: the intervening attenuations are indicated by fractions, of which the numerator is an Arabic, and the denominator a Roman numeral; thus, $\frac{1}{\text{I}}$ means the fourth, $\frac{2}{\text{I}}$ the fifth, and $\frac{1}{\text{II}}$ the seventh attenuation.

In computing the value of these homœopathic fractions, the reader must recollect, that each unit of the Roman numerals stands for three attenuations, but each Arabic unit for one only: thus $\frac{1}{\text{VI}}$ will denote nineteen attenuations.

We now take leave of our author, giving him our hearty thanks for his amusing book; indeed, his work is more than amusing; for, although the thinking physician will not adopt Dr. Quin's practice, he will certainly improve his own by the reflections which spring from the consideration of homœopathy.

